# Intranasal immunization with peptide-based immunogenic complex enhances BCG vaccine efficacy in a murine model of tuberculosis

**DOI:** 10.1172/jci.insight.145228

**Published:** 2021-02-22

**Authors:** Santosh Kumar, Ashima Bhaskar, Gautam Patnaik, Chetan Sharma, Dhiraj Kumar Singh, Sandeep Rai Kaushik, Shivam Chaturvedi, Gobardhan Das, Ved Prakash Dwivedi

**Affiliations:** 1 International Centre for Genetic Engineering and Biotechnology (ICGEB), New Delhi, India.; 2 Signal Transduction Laboratory-1, National Institute of Immunology, New Delhi, India.; 3 Special Center for Molecular Medicine, Jawaharlal Nehru University, New Delhi, India.

**Keywords:** Immunology, Infectious disease, Tuberculosis

## Abstract

Prime-boost immunization strategies are required to control the global tuberculosis (TB) pandemic, which claims approximately 3 lives every minute. Here, we have generated an immunogenic complex against *Mycobacterium tuberculosis* (*M.tb*), consisting of promiscuous T cell epitopes (*M.tb* peptides) and TLR ligands assembled in liposomes. Interestingly, this complex (peptide–TLR agonist–liposomes; PTL) induced significant activation of CD4^+^ T cells and IFN-γ production in the PBMCs derived from PPD^+^ healthy individuals as compared with PPD^–^ controls. Furthermore, intranasal delivery of PTL significantly reduced the bacterial burden in the infected mice by inducing *M.tb*-specific polyfunctional (IFN-γ^+^IL-17^+^TNF-α^+^IL-2^+^) immune responses and long-lasting central memory responses, thereby reducing the risk of TB recurrence in DOTS-treated infected animals. The transcriptome analysis of peptide-stimulated immune cells unveiled the molecular basis of enhanced protection. Furthermore, PTL immunization significantly boosted the Bacillus Calmette-Guerin–primed (BCG-primed) immune responses against TB. The greatly enhanced efficacy of the BCG-PTL vaccine model in controlling pulmonary TB projects PTL as an adjunct vaccine against TB.

## Introduction

*Mycobacterium tuberculosis* (*M.tb*), the causative agent of tuberculosis (TB), affects about one-fourth of the global population (1). Approximately 2 million deaths globally are directly attributed to TB. Synergism between HIV infections and *M.tb*, along with the emergence of multidrug-resistant strains of *M.tb*, has become a major concern for nations globally (2, 3). Unfortunately, cost-effective and user-friendly therapy for TB infections is long overdue. *M.tb* infections may produce varied responses between the individuals, ranging from asymptomatic infections to progressive pulmonary or extrapulmonary TB — and even death (4). The rate of progression in the severity of TB depends on the status of the host immune system.

Although the world’s only accepted vaccine against TB, the live attenuated strains of *Mycobacterium bovis* Bacillus Calmette-Guerin (BCG) is very effective against disseminated and meningeal TB in young children. However, its efficacy in protecting against adult pulmonary TB varies dramatically from 0%–80% in different populations depending upon ethnicity and geographical regions (5–9). BCG’s limited vaccine efficacy is majorly attributed to its failure to induce a significant population of central memory T cells (Tcm) (6, 9–11), since animal models vaccinated with BCG primarily develop antigen-specific CD4^+^ effector memory T cells (Tem). Considering the lags in BCG immunization and increased global TB burden, it is crucial to develop improved methods of immunoprophylaxis against TB. Since most of the world’s population is vaccinated with BCG, we need an alternative therapy to improve the efficacy of BCG in terms of enhancing central memory responses leading to the induction of polyfunctional cytokine responses at the site of infection, eventually controlling the infection.

Surface antigens, along with the secretome of mycobacteria, have been shown to generate potent host immune responses during *M.tb* infection (12–15). Taking a cue from above findings, in this study, we generated an immunogenic complex against *M.tb*, which consisted of promiscuous protective T cell epitopes along with TLR ligands adsorbed on liposomal drug delivery vehicle. These complexes, called peptide–TLR agonist–liposomes (PTL), were delivered directly into the lungs through an intranasal route, thereby generating a protective immune response at the site of infection.

We observed that the PTL significantly enriched the BCG-induced Tcm pool in the CD4^+^ and CD8^+^ T cells, with a decrease in the Tem cell pool in the lungs of mice coimmunized with BCG and PTL, compared with the mice immunized with BCG or PTL alone. The population of Tcm was maintained at elevated numbers in the spleens of coimmunized animals, as well, consistent with the understanding that spleens are the potential reservoir of these cells (9). Interestingly, the frequency of immunosuppressive PD-1 expression on memory cell subsets was significantly low in the lungs and spleens of BCG and PTL coimmunized mice as compared with other groups. Moreover, increased memory responses correlated with a remarkable reduction in bacterial burden in the lungs, spleens, and livers of the animals receiving PTL immunization along with BCG compared with other experimental groups.

Furthermore, we also noticed a significant increase in the polyfunctional cytokine secretion (IFN-γ^+^IL-17^+^TNF-α^+^IL-2^+^) in CD4^+^ and CD8^+^ T cells in the lungs of coimmunized animals as compared with the mice vaccinated with BCG alone. A similar protective response was also observed in reactivation studies. Separate transcriptome analysis of DCs pulsed with peptides (Pep-DCs) and cocultured T cells further sheds light on the possible multiple host-protective pathways induced by PTL.

Collectively, in our study, we report that BCG-vaccinated mice, when coimmunized with PTL, induced a larger pool of Tcm cells, which may contribute to a stronger and a potent recall immune response to facilitate enhanced *M.tb* clearance. In brief, our findings suggest that PTL coimmunization in BCG-vaccinated mice significantly enhances the vaccine efficacy of BCG.

## Results

### T cell peptides derived from M.tb induce host-protective immune responses.

**Figure 1:**
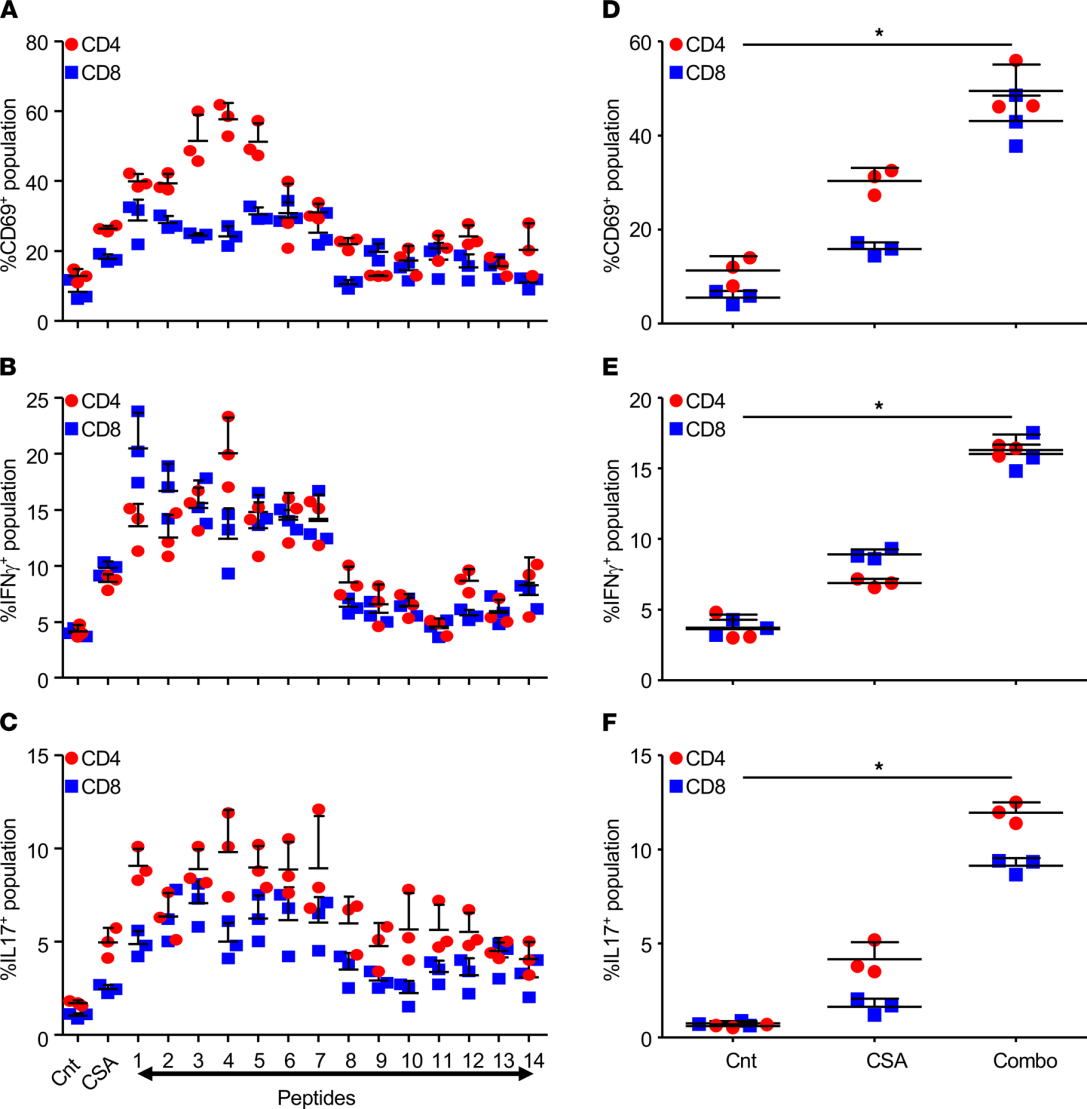
Mycobacterial antigens induce activation of protective T cell responses. T cells isolated from the spleens of mice infected with H37Rv and treated with DOTS were stimulated with DCs preloaded with the peptides for 48 hours, followed by surface staining with anti-CD3, anti-CD4, anti-CD8, and anti-CD69 and staining for intracellular cytokines with anti–IFN-γ and anti–IL-17 antibodies. (**A**–**C**) Bar graphs depicting the percentage of CD4^+^CD69^+^ and CD8^+^CD69^+^ T cells (**A**), CD4^+^IFN-γ^+^ and CD8^+^IFN-γ^+^ T cells (**B**), and CD4^+^IL-17^+^ and CD8^+^IL-17^+^ T cells (**C**). (**D**) Activation of CD4^+^ and CD8^+^ T cells cocultured with unstimulated DCs (Cnt), DCs pulsed with CSA, or DCs pulsed with the peptide combo. (**E** and **F**) CD4^+^ and CD8^+^ T cells expressing IFN-γ (**E**) and ΙL-17 (**F**) after stimulation with the combo. Each experiment was performed at least three times in triplicate. Two-tailed Student’s *t* test was performed for statistical analysis. Data represent mean ± SD (*n* = 3). *P < 0.05.

*M.tb* peptides derived from ESAT6, Ag85B, and MPT70 have been shown to be promising candidates for the induction of protective T cell responses during TB. Taking observations from the previous studies, we screened 14 *M.tb* peptides derived from different secretory proteins of H37Rv for their efficacy to induce *M.tb*-specific T cell activation and host-protective Th1/Th17 responses (Supplemental Table 1; supplemental material available online with this article; https://doi.org/10.1172/jci.insight.145228DS1) (12–16). A group of mice infected with the H37Rv strain of *M.tb* was subjected to 45 days of DOTS therapy starting from 15 days after infection. After a rest period of 30 days, T cells from these infected and DOTS-treated mice were isolated and cocultured with DCs derived from the BM of naive mice and pulsed with T cell epitopes/peptides (0.2 μg/mL) or complete soluble antigen (CSA) of *M.tb* (20 μg/mL). From these sets of peptides, 7 peptides — which induced significant T cell activation (Figure 1A) and enhanced IFN-γ and IL-17 secretion (Figure 1, B and C) — are indicated in Supplemental Table 2. Next, we performed the above experiments using a pool of these 7 antigenic peptides (combo, 100 ng/mL of each peptide) and CSA of *M.tb* as a positive control. Combo significantly increased the expression of early surface activation marker CD69 on CD4^+^ cells and CD8^+^ T cells (Figure 1D) in comparison with CSA of *M.tb*. Furthermore, we also observed an increase in the number of IFN-γ– and IL-17–producing CD4^+^ and CD8^+^ T cells (Figure 1, E and F) in the T cell pool cocultured with DCs loaded with peptide pool in comparison with the CSA.

### M.tb peptides induce the gene expression signature required for protective immunity in DCs and T cells.

**Figure 2:**
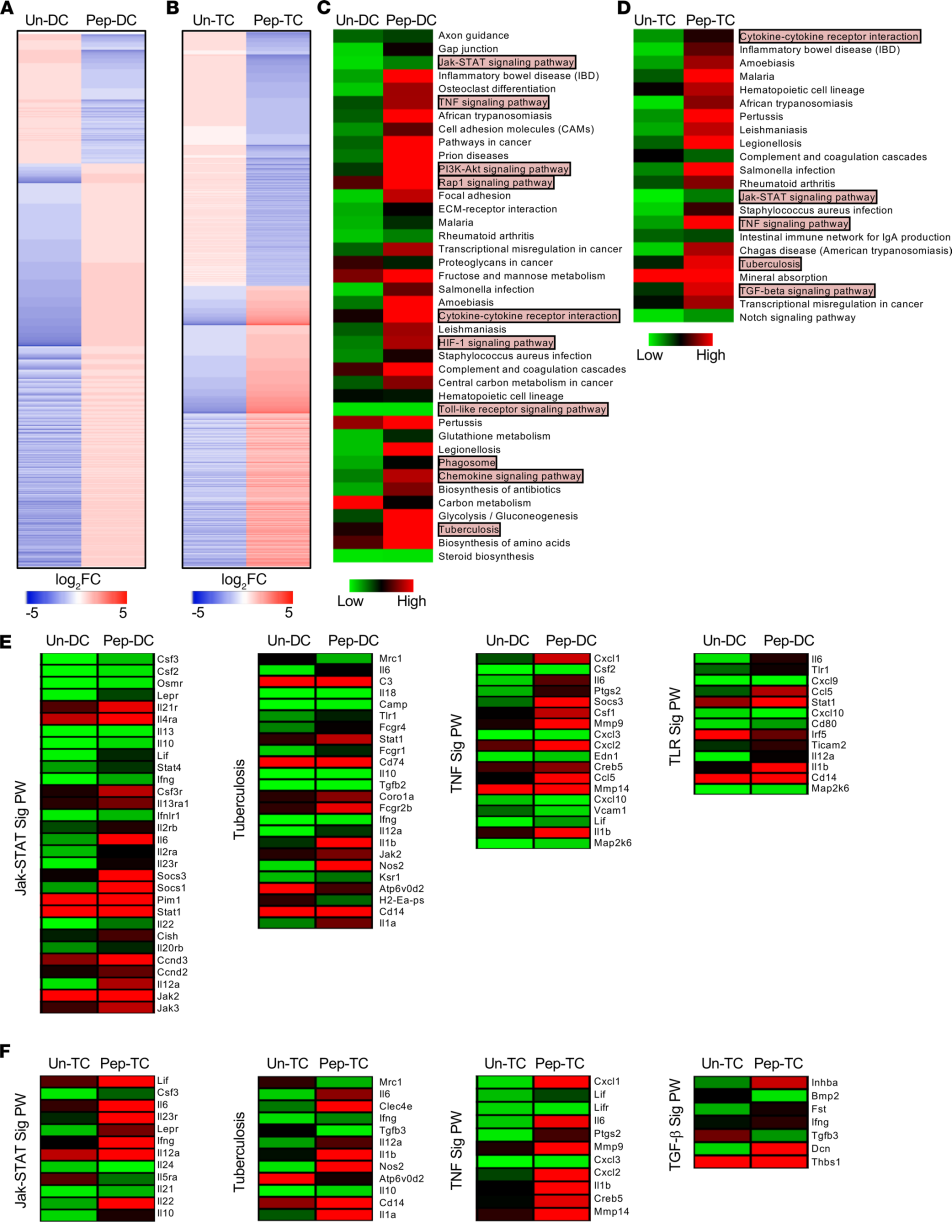
Stimulation with peptide pool induces multiple signaling pathways involved in providing protection during *M.tb* infection. (**A** and **B**) Heatmap representation of the genes differentially expressed in the DCs pulsed with peptide pool (Pep-DC) in comparison with unstimulated DCs (Un-DC) (**A**) and the T cells cocultured with DCs pulsed with peptide pool (Pep-TC) versus T cells cocultured with unstimulated DCs (Un-TC) (**B**). Red depicts activation, while blue represents repression. (**C**) Molecular signaling pathways majorly affected in Pep-DCs. (**D**) KEGG pathways significantly modulated in Pep-TCs. (**E**) Heatmaps representing the JAK/STAT signaling pathway, TB, TNF signaling pathway, and TLR signaling pathways in Pep-DCs versus Un-DCs. (**F**) Heatmaps representing the JAK/STAT signaling pathway, TB, TNF signaling pathway, and TGF-β signaling pathways in Pep-TCs versus Un-TCs. RNA-Seq was performed once in triplicate (*n* = 3).

To further understand the host changes at the transcription levels leading to an increased T cell activation and an augmented proinflammatory cytokine upon peptide pool treatment, we performed comparative transcriptome analysis of unstimulated DCs (Un-DCs) versus Pep-DCs and T cells cocultured with unstimulated DCs (Un-TCs) versus T cells cocultured with DCs pulsed with peptides (Pep-TCs). Our RNA sequencing (RNA-Seq) data revealed 1452 differentially expressed genes (1098 upregulated, log_2_FC > 1; 354 downregulated, log_2_FC < –1 with FDR ≤ 0.05) in the peptide pool–stimulated DCs as compared with Un-DCs (Figure 2A), while Pep-TCs showed 2331 differentially expressed genes (1223 activated and 1108 repressed) as compared with Un-TCs (accession no. GSE164258) (Figure 2B). Differential genes in both DCs and T cells after peptide pool stimulation were highly enriched for gene sets assisting in IFN-γ response and production, cytokine activity, STAT phosphorylation, ADP metabolic processes, and ROS equilibrium (Supplemental Figure 1, A and B). Many pathways known to play an important role in combating TB disease were significantly upregulated in peptide-stimulated DCs and T cells, as indicated by KEGG analysis (Figure 2, C and D). Differential genes in the DCs as well as the T cells majorly belonged to signaling pathways such as JAK/STAT, TB, TNF, TLR, and TGF-β signaling (Figure 2, E and F). The transcriptome data analysis revealed a very similar and indistinguishable trend of activated genes in DCs and T cells. A huge number of 724 genes (586 upregulated and 138 downregulated) were common between the 2 cell types (Supplemental Figure 1C). Moreover, these genes followed the same expression profile in both of the settings (Supplemental Figure 1C). KEGG analysis indicated that these genes belonged to a number of TB-related protective host signaling pathways such as NF-κB, MAPK, TGF-β, TNF, and IL-17 (Supplemental Figure 1D). Taken together, our transcriptomics data have strengthened our findings that peptide pool induces an intricate network of signaling pathways in DCs and T cells, which leads to enhanced cytokine production and T cell activation.

### Induction of immune responses in human PBMCs by the M.tb PTL assembly.

**Figure 3:**
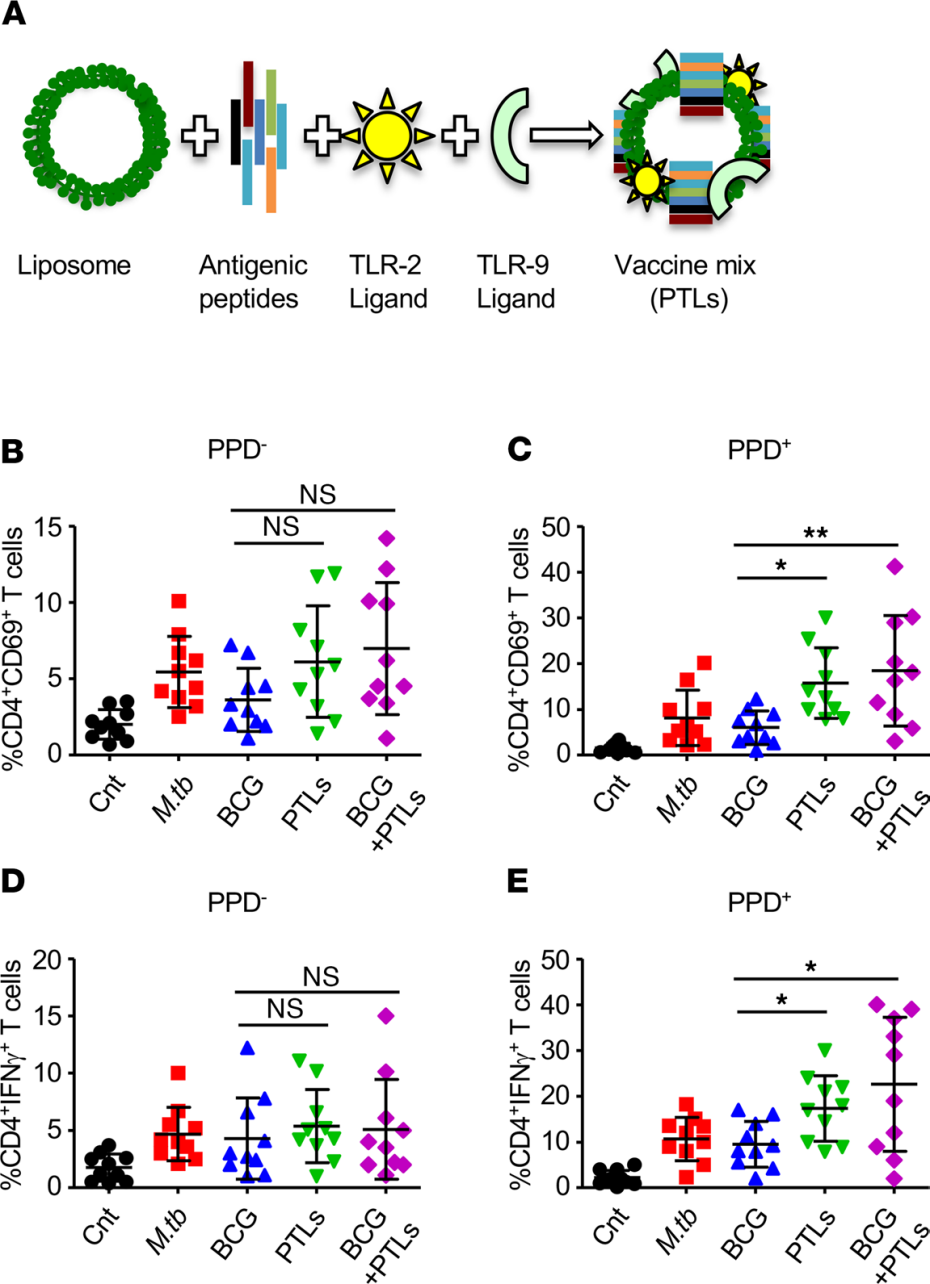
PTL induces a protective immune response in human PBMCs. (**A**) Schematic diagram depicts the preparation of PTL. PBMCs isolated from PPD^–^ and PPD^+^ healthy subjects were in vitro stimulated with different mycobacterial antigens for 48 hours. (**B** and **C**) Expression of CD69^+^ on CD4^+^ T cells stimulated with mycobacterial antigens in PPD^–^ (**B**) and PPD^+^ (**C**) individuals. (**D** and **E**) Percentage of CD4^+^ T cells expressing IFN-γ in the PBMCs of PPD^–^ (**D**) and PPD^+^ (**E**) individuals. One-way ANOVA, followed by multiple Tukey tests, was performed for statistical analysis. Data represent mean ± SD (*n* = 10) performed once. *P < 0.05, **P < 0.005.

Subject to the above results, we establish here that, in combination, our peptides are capable of inducing antigen-specific protective T cells; therefore, we assembled these 7 antigenic peptides — along with TLR2 and TLR9 agonist Pam3CysSK-4 and CpG ODN, respectively, as evidenced by previous reports that TLR2 and TLR9 play an important role during *M.tb* infection (17, 18) — in a liposomal delivery vehicle for the successful delivery of this cargo to the lungs through the intranasal route (Figure 3A). The efficacy of this assembly of PTL was assessed in human PBMCs derived from 10 PPD^–^ and 10 PPD^+^ BCG vaccinated healthy individuals. The PBMCs were in vitro stimulated with *M.tb* CSA (20 μg/mL), BCG CSA (20 μg/mL), PTL (10 μL/mL), and BCG CSA/PTL for 48 hours, followed by surface/intracellular staining and FACS analysis, to determine CD4^+^ T cell activation and IFN-γ production. With no significant difference in PPD^–^ individuals, PTL and BCG CSA/PTL combination induced significant expression of early activation marker CD69 and IFN-γ on CD4^+^ T cells in the PBMCs derived from PPD^+^ individuals as compared with *M.tb* CSA and BCG CSA controls (Figure 3, B–E).

### PTL immunization following BCG vaccination enhances host protection against TB.

**Figure 4:**
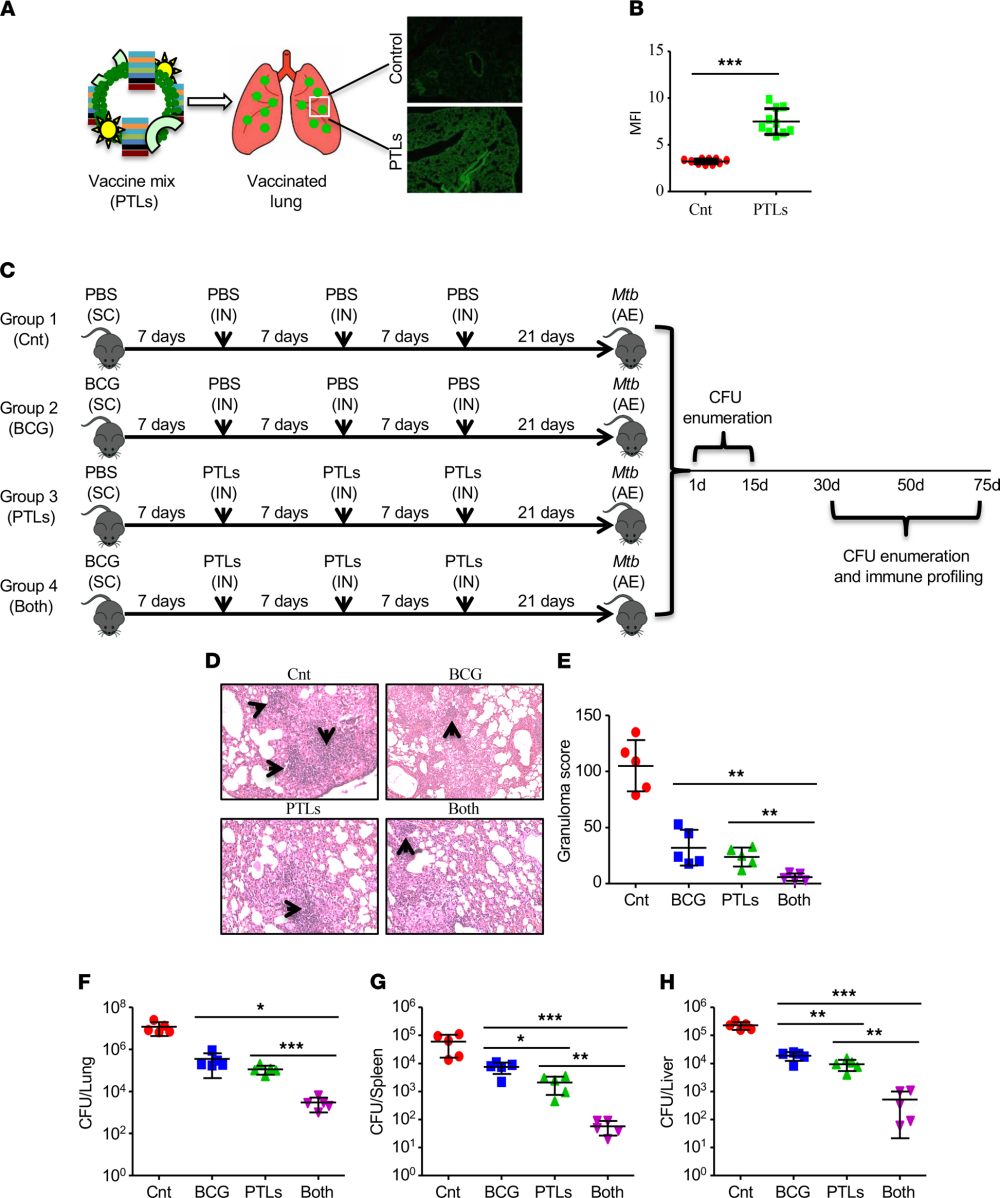
PTL enhances the efficacy of BCG and protects mice against TB. (**A**) Lung section to show the accumulation of liposomes. Liposomes were stained with PKH67 dye. Original magnification, ×100. (**B**) Quantification of the fluorescent images. (**C**) Layout to show the experimental plan wherein naive C57BL/6 mice or mice vaccinated with BCG/PTL or a combination of both were challenged with H37Rv via the aerosol route with a low-dose inoculum of ~150 CFU/mice. Mice were sacrificed at various time points, and lungs, spleen, and liver were harvested for observance of bacterial burden, as well as profiling of immune responses. (**D**) Lungs were harvested, preserved in 4% paraformaldehyde, and processed for sectioning and staining with H&E. Original magnification, ×100. (**E**) Quantification of the granuloma (inflammatory lesions) in all experimental groups. (**F**–**H**) CFU from the lung (**F**), spleen (**G**), and liver homogenates (**H**) at 50 days after infection. Two-tailed Student’s *t* test was performed for statistical analysis. Data are representative of 2 independent experiments (*n* = 5 mice/group). *P < 0.05, **P < 0.005, ***P < 0.0005.

To confirm the successful delivery of this assembly of PTL into the lungs, the PTL were labeled with PKH67 to stain the liposomes (delivery vehicle) and administered intranasally into the lungs. Two days after delivery, mice were euthanized and lung sections were prepared for fluorescence microscopy (Figure 4A). Quantification of the fluorescent images indicated the accumulation of liposomes/PTL throughout the lungs (Figure 4B).

Despite its limited efficacy in adults, BCG vaccine is highly successful in young children and, as a result, is administered in infants and small children in high-burden countries. Keeping in mind the existing load of the global population vaccinated with BCG, we designed a strategy wherein BCG-vaccinated mice were boosted with a once-a-week, 3-week PTL boosting regimen followed by a rest period of 21 days. Supplemental Figure 2 shows the prechallenge immune response in the lungs and the spleens of vaccinated animals. With no increase in the number of CD4^+^ and CD8^+^ T cells, we observed a significant increase in the expression of CD69 on these cells in the lungs of BCG-PTL–coimmunized mice as compared with BCG/PTL administration alone (Supplemental Figure 2, A–D). Furthermore, coimmunized animals also showed significant increase in the CD4^+^ T cells expressing IFN-γ and IL-17 (Supplemental Figure 2, E and F) as compared with single vaccinations. Similarly, CD8^+^ T cells expressing IL-17 but not IFN-γ were also induced in the lungs of coimmunized mice (Supplemental Figure 2, G and H). A similar profile was observed in the spleens of vaccinated animals (Supplemental Figure 2, I–P). These mice were challenged with H37Rv, the laboratory strain of *M.tb*, using low-dose aerosol infection model (approximately 150 CFU/mice), after which organs were harvested at different time points to look at the bacterial burden, along with the elicited immune responses (Figure 4C). Consistent with our expectations, coimmunized animals had fewer and smaller inflammatory lesions in their lungs than mice immunized with BCG or PTL alone, while the nonvaccinated infected mice (i.e., primary infection with H37Rv *M.tb*) showed a significantly higher number of inflammatory lesions (*P* < 0.005, Figure 4D). These results were further strengthened by histopathological analysis of lungs, which confirmed the reduced lung inflammation in the coimmunized group as compared with the rest of the experimental groups (Figure 4E). PTL coimmunization significantly increased the BCG-induced TB protection, as observed by the reduced bacterial burden in various organs, such as the lungs (Figure 4F), spleens (Figure 4G), and livers (Figure 4H) of infected mice. Thus, the PTL significantly enhanced the antitubercular capacity of BCG immunization. Interestingly, the mice immunized with PTL alone displayed comparable (Figure 4F) and enhanced (Figure 4, G and H) resistance against *M.tb* as the BCG immunized group.

### BCG-PTL coimmunization elevates the adaptive immunity in the lungs and the spleens M.tb-infected mice.

**Figure 5:**
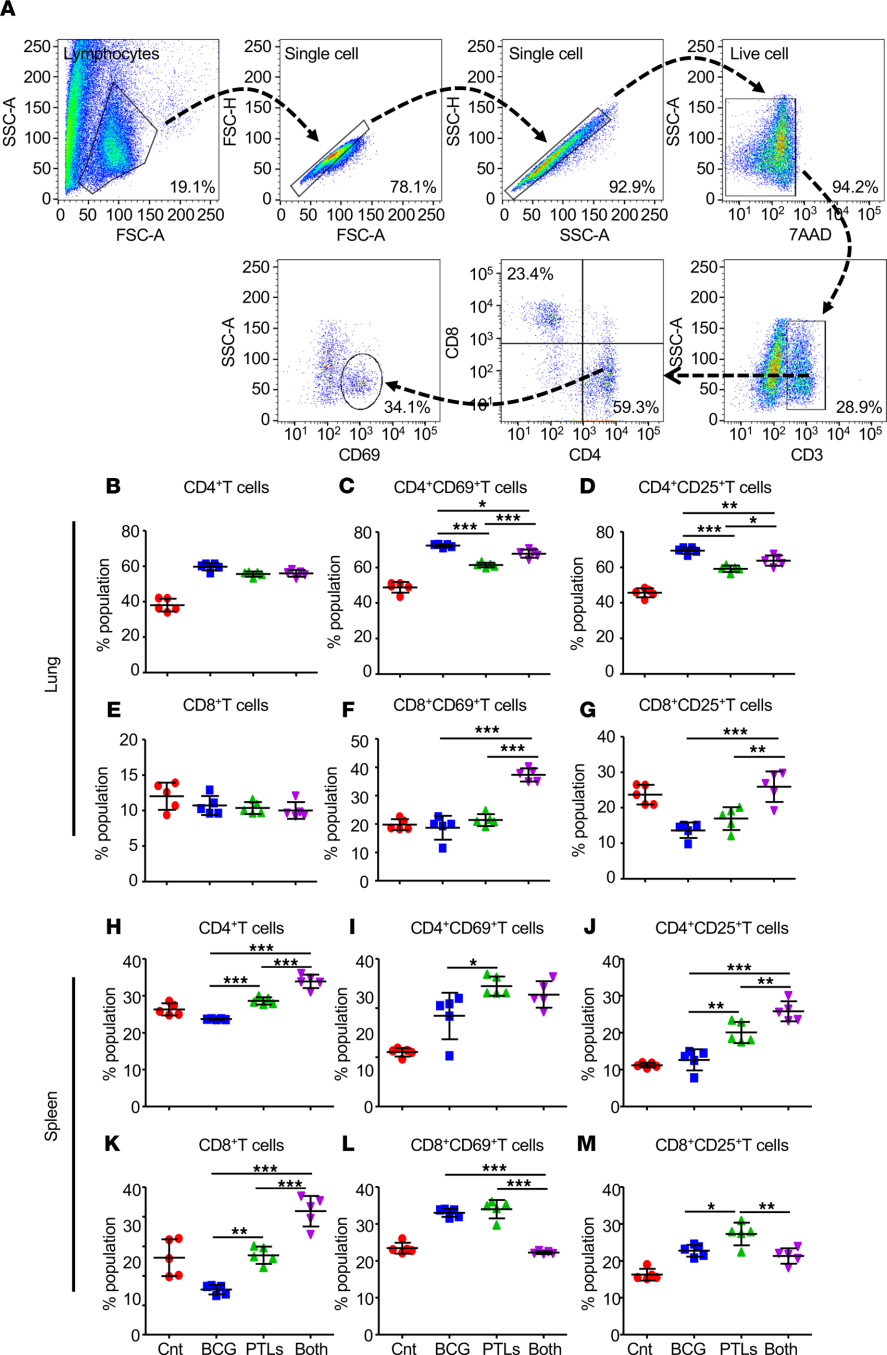
PTL immunization induces T cell activation in the lungs and the spleens of infected animals. T lymphocytes were isolated from the lungs of all experimental groups and stained with 7AAD, anti-CD3, anti-CD4, anti-CD8, anti-CD25, and anti-CD69 antibodies. (**A**) Gating strategy employed to quantify the T cell activation. (**B**–**D**) Percentage of CD4^+^ T cells (**B**) and expression of CD69 (**C**) and CD25 (**D**) on CD4^+^ T cells in the lungs of infected animals. (**E**–**G**) Percentage of CD8^+^ T cells (**E**) and expression of CD69 (**F**) and CD25 (**G**) on CD8^+^ T cells in the lungs of infected animals. (**H**–**M**) T lymphocytes were isolated from the spleens of all experimental groups and stained with 7AAD, anti-CD3, anti-CD4, anti-CD8, anti-CD25, and anti-CD69 antibodies. (**H**–**J**) Percentage of CD4^+^ (**H**), CD4^+^CD69^+^ (**I**), and CD4^+^CD25^+^ (**J**) T cells in the spleens of infected animals. (**K**–**M**) Percentage of CD8^+^ (**K**), CD8^+^CD69^+^ (**L**), and CD8^+^CD25^+^ (**M**) T cells in the spleens of infected animals. One-way ANOVA, followed by multiple Tukey tests, was performed for statistical analysis. Data are representative of 2 independent experiments (*n* = 5 mice/group). *P < 0.05, **P < 0.005, ***P < 0.0005.

The host immune system plays a pivotal role in defending against TB pathogenesis. To delve into the immunological changes involved in enhanced protection conferred by BCG-PTL coimmunization, we profiled the immune cells in the lungs and the spleens of infected animals. Figure 5A describes the gating strategy employed to quantify the percentage of various T cell subsets in the lungs and spleens of infected mice. BCG-PTL–coimmunized mice showed comparable levels and activation of CD4^+^ (Figure 5, B–D), whereas with minimal effect on the percentage of CD8^+^ T cells (Figure 5E), BCG-PTL coimmunization significantly enhanced the expression of early and late activation markers (CD69 and CD25, respectively) on CD8^+^ T cells as compared with either BCG or PTL immunization (Figure 5, F and G). Furthermore, we observed increased frequency of CD4^+^ T cells (Figure 5H) with increased expression of late activation marker CD25 in the spleens of BCG-PTL–coimmunized animals (Figure 5J). CD69 expression was significantly high in splenic CD4^+^ T cells derived from PTL-immunized animals as compared with BCG group (Figure 5I). Percentage of CD8^+^ T cells was significantly high in the spleens of BCG-PTL–immunized animals (Figure 5K) with no increase in the early and late activation markers (Figure 5, L and M).

### BCG-PTL coimmunization induces polyfunctional cytokine responses in the lungs and the spleens of M.tb-infected mice.

**Figure 6:**
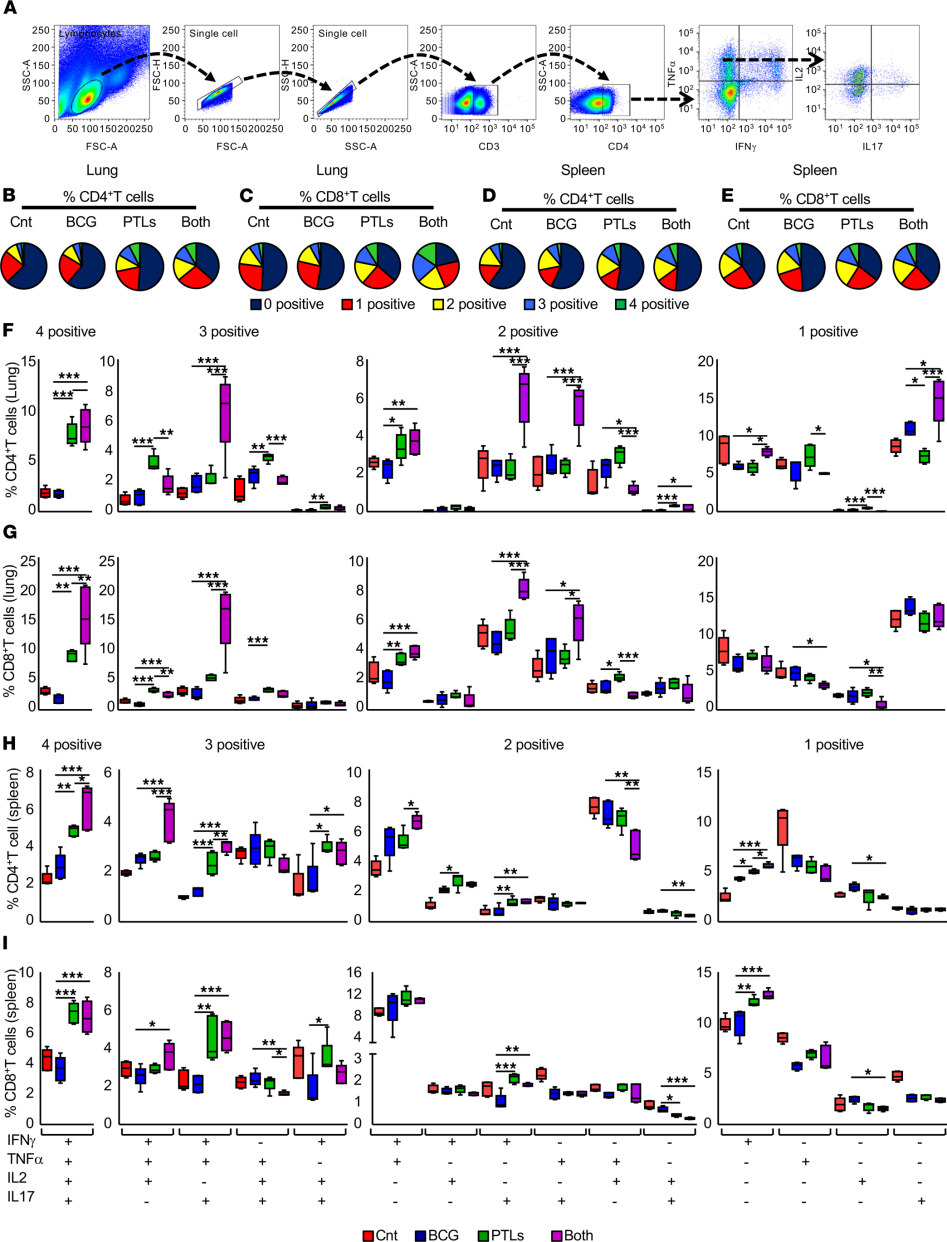
BCG-PTL coimmunization induces the antigen-specific polyfunctional cytokine responses in the lungs and the spleens of infected animals. (**A**) Lymphocytes isolated from the lungs of infected animals were stained with anti-CD3, anti-CD4, anti-CD8, anti–IFN-γ, anti-TNF-α, anti–IL-17, and anti–IL-2 to assess polyfunctional cytokine responses. (**B** and **C**) Pie charts depicting the percentage of CD4^+^ (**B**) and CD8^+^ (**C**) T cells expressing 4, 3, 2, 1, and 0 cytokines (IFN-γ, TNF-α, IL-17, and IL-2) in the lungs of the mice. (**D** and **E**) The pie charts representing the average percentage of cytokine-producing CD4^+^ (**D**) and CD8^+^ (**E**) T cells producing 5 combinations (0^+^, 1^+^, 2^+^, 3^+^, and 4^+^) of the 4 cytokines analyzed in the spleens of infected animals. (**F** and **G**) Fifteen possible cytokine combinations are shown for CD4^+^ (**F**) and CD8^+^ (**G**) T cells from the lungs of infected animals. (**H** and **I**) Box and whisker plots depict 15 combinations of responses for the 4 cytokines analyzed on the *x* axis with the percentage of CD4^+^ (**H**) and CD8^+^ (**I**) responding splenic T cells on the *y* axis. One-way ANOVA, followed by multiple Tukey tests, was performed for statistical analysis. Data are representative of 2 independent experiments (*n* = 5 mice/group). *P < 0.05, **P < 0.005, ***P < 0.0005.

To investigate the T cell–specific cytokine responses in different experimental groups, we isolated the T cells from the lungs and the spleens of treated and control mice and estimated intracellular cytokine production. Polyfunctional T cells expressing more than 2 cytokines, such as IFN-γ, TNF-α, IL-17, and IL-2, have been linked with enhanced protection against TB (19, 20). Moreover, the occurrence of these multifunctional T cells is highly important in the context of effective vaccines against various viruses and intracellular bacterial pathogens (21). Thus, we analyzed the presence of polyfunctional cells in different T cell subsets in the lungs and the spleens of all animal groups (Figure 6A). Interestingly, BCG-PTL coimmunization significantly increased the frequency of CD4^+^ and CD8^+^ T cells expressing single, double, triple, and quadruple cytokines, with a concomitant decrease in the T cell population expressing none of the 4 cytokines in the infected lungs (Figure 6, B and C) and the spleens (Figure 6, D and E) as compared with other groups. Particularly, there was an increased occurrence of 4-positive (IFN-γ^+^TNF-α^+^IL-17^+^IL-2^+^), 3-positive (IFN-γ^+^TNF-α^+^IL-17^+^), and 2-positive (IFN-γ^+^IL-17^+^ and TNF-α^+^IL-17^+^) CD4^+^ and CD8^+^ T cells in the coimmunized group as compared with other animals (Figure 6, F and G). However, the expression of single cytokines (IFN-γ^+^ and IL-17^+^) was higher only in CD4^+^ T cells (Figure 6, F and G). A similar profile was observed in the spleens of coimmunized mice where 4-positive (IFN-γ^+^TNF-α^+^IL-17^+^IL-2^+^), 3-positive (IFN-γ^+^TNF-α^+^IL-17^+^;IFN-γ^+^TNF-α^+^IL-2^+^), 2-positive (IFN-γ^+^IL-17^+^), and 1-positive (IFN-γ^+^) CD4^+^ and CD8^+^ T cells displayed significantly enhanced frequency in BCG-PTL–coimmunized group (Figure 6, H and I). Taken together, we observed that BCG vaccination followed by PTL immunization greatly enhanced the antigen-specific Th1 and Th17 responses, as well as proinflammatory cytokines TNF-α and IL-2. All these cytokines have been well documented to impart protection against TB.

### PTL immunization induces Tcm cell responses critical for long-lasting protection.

**Figure 7:**
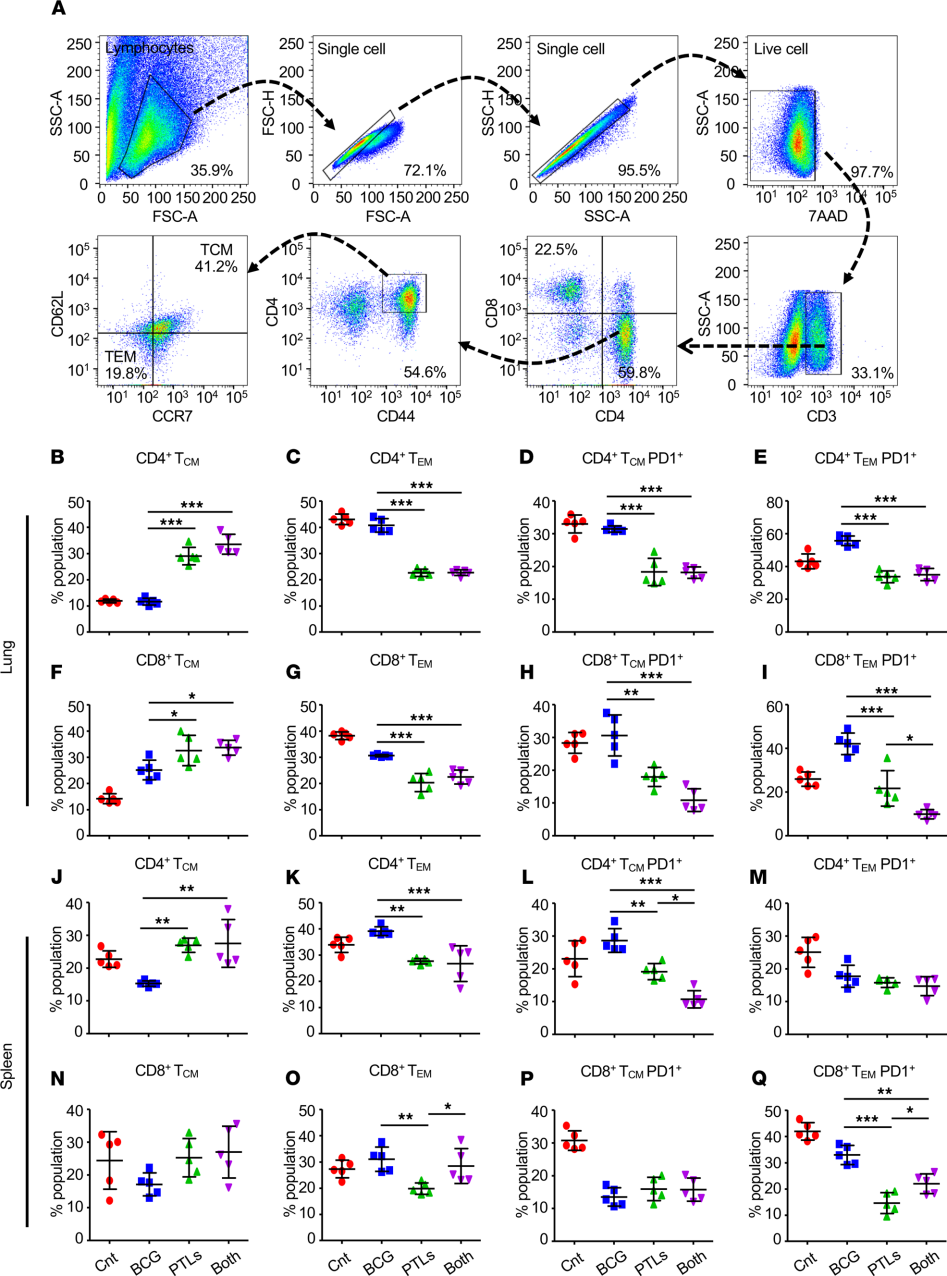
PTL induces superior antigen-specific T cell memory responses in the lungs and the spleens of infected mice. T lymphocytes isolated from the lungs and the spleens of the indicated groups of experimental mice at 50 days after infection were surface stained with anti-CD3, anti-CD4, anti-CD8, anti-CCR7, anti-CD44, anti-CD62L, and anti–PD-1 antibodies and fixed prior to acquisition by flow cytometry. (**A**) Gating strategy employed to quantify the memory T cell responses. (**B** and **C**) Percentage of central memory (Tcm; CCR7^hi^CD62L^hi^CD44^hi^) (**B**) and effector memory (Tem; CCR7^lo^CD62L^lo^CD44^hi^) (**C**) CD4^+^ T cells on lymphocytes isolated from the lungs of infected animals. (**D** and **E**) Frequency of PD-1 expression on central memory (**D**) and effector memory (**E**) CD4^+^ T cell subset. (**F** and **G**) Percentage of Tcm (CCR7^hi^CD62L^hi^CD44^hi^) (**F**) and Tem (CCR7^lo^CD62L^lo^CD44^hi^) (**G**) CD8^+^ T cells on lymphocytes isolated from the lungs of infected animals. (**H** and **I**) Frequency of PD-1 expression on these cell subsets. (**J**–**Q**) Frequency of central memory, effector memory, and PD-1 expression on these cell subsets on the lymphocytes isolated from the spleens of infected mice. One-way ANOVA, followed by multiple Tukey tests, was performed for statistical analysis. Data are representative of 2 independent experiments (*n* = 5 mice/group). *P < 0.05, **P < 0.005, ***P < 0.0005.

Since recall responses are mediated by Tcm cells, these cell subtypes become a prerequisite for enhanced vaccine efficacy and superior TB protection (6, 9, 22). Thus, we analyzed the memory cell profile of the lung, as well as the splenic T cell subsets (Figure 7A). We observed that BCG-PTL coimmunization enriched the vaccine-induced Tcm (CD44^hi^CCR7^hi^CD62L^hi^) cell pool in CD4^+^ T cells (Figure 7B) with a concomitant decrease in the Tem (CD44^hi^CCR7^lo^CD62L^lo^) cell pool (Figure 7C) in the lungs of infected mice as compared with BCG-vaccinated animals. Expression of inhibitory receptors such as PD-1 and CTLA-4 is often linked with negative regulation and inhibition of activated T cells, leading to T cell exhaustion (23, 24). Moreover, in nonhuman primates and in several human studies, increased PD-1 expression is linked with severe TB pathology and enhanced bacillary load (25, 26). Interestingly, decreased expression of PD-1 was observed on the CD4^+^ Tcm and Tem subsets in the lungs of mice coimmunized with BCG and PTL as compared with the BCG-vaccinated group (Figure 7, D and E). A similar pattern was observed in the CD8^+^ lung T cells (Figure 7, F–I). When investigated in the spleens of coimmunized animals, there was increased frequency of CD4^+^ Tcm pool (Figure 7J) with a decrease in CD4^+^ Tem subset (Figure 7K). PD-1 expression was significantly less only in the case of the CD4^+^ Tcm subset (Figure 7L) in the coimmunized group, with no effect on the CD4^+^ Tem subset (Figure 7M). CD8^+^ Tcm/Tem cells were comparable in all the groups (Figure 7, N and O). With no decrease in PD-1 expression on CD8^+^ Tcm (Figure 7P), its expression was significantly less on the CD8^+^ Tem subset in the coimmunized group (Figure 7Q). Previously, it has been shown that BCG-vaccinated or *M.tb*-infected mice generate a profoundly expanded population of antigen-specific Tem cells within the lungs, whereas the Tcm pool is substantially smaller. However, Tcm cells are maintained in significantly larger numbers in the spleen, which is believed to be a potential reservoir for these cells (9, 22). While we found similar Tcm/Tem profiles for infected and BCG-vaccinated controls, coimmunized mice maintained an increased pool of Tcm cells both in the spleen and the lungs. Nuclear FOXO1 is in an unphosphorylated state and keeps the long-lived memory T cells enriched while the phosphorylated FOXO1 protein leaves the nucleus and is tagged for ubiquitin-mediated protein degradation (27, 28). Interestingly, we also observed a significant reduction in the phosphorylation of FOXO1 transcription factor in the splenocytes of coimmunized animals in comparison with all other experimental groups (Supplemental Figure 3). Furthermore, we observed an increased activation of NF-κB transcription factor in the splenocytes of BCG-PTL–coimmunized animals as compared with other groups (Supplemental Figure 3). NF-κB is believed to be the main transcription factor responsible for the expression of various proinflammatory cytokines, such as IFN-γ, TNF-α, and IL-12, required for providing resistance to TB. Collectively, these data suggest that PTL used in this study induces the activation of key transcription factors involved in generating protective immune responses inside the host.

### Antigen-specific protective immunity induced by BCG-PTL coimmunization can be adoptively transferred by T cells.

**Figure 8:**
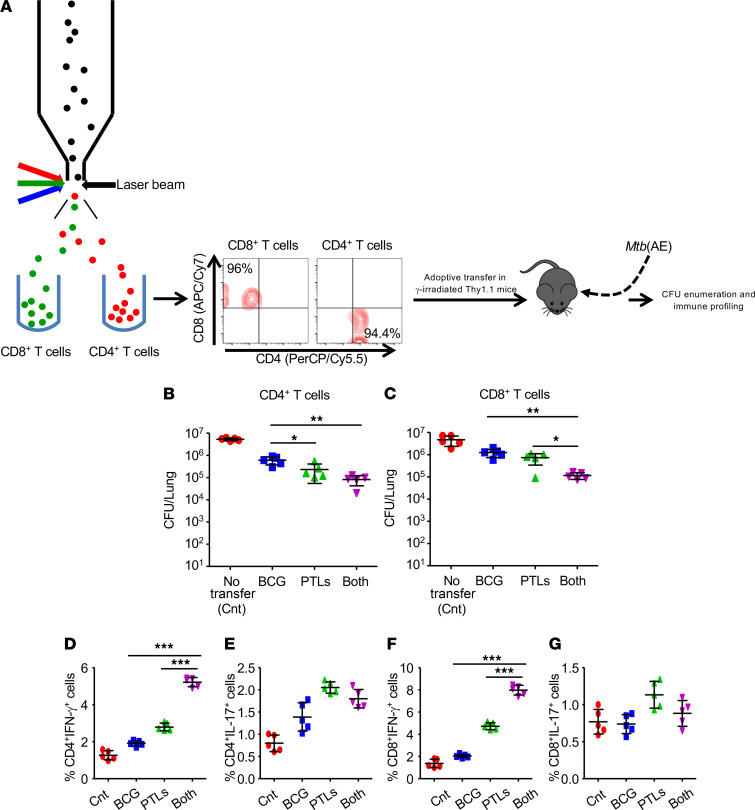
T cells from BCG-PTL–coimmunized mice confer improved protection against TB. (**A**) T lymphocytes isolated from BCG, PTL, or BCG-PTL immunized and *M.tb*-infected animals (50 days after infection) were subjected to surface staining with anti-CD3, anti-CD4, and anti-CD8 followed by sorting by FACSAria into 2 distinct populations: CD4^+^ and CD8^+^ T cells. Sorted CD4^+^ and CD8^+^ T cells were cultured overnight and transferred into irradiated recipient Thy1.1 mice. Seven days after adoptive transfer, all mice were challenged with *M.tb* H37Rv through the aerosol route. At 25 days after infection, the mice were euthanized for CFU enumeration and immune profiling. (**B** and **C**) CFU in the lungs of mice receiving CD4^+^ (**B**) and CD8^+^ (**C**) T cells. (**D**–**G**) Dot plots representing the percentage of INF-γ– and IL-17–producing CD4^+^ and CD8^+^ T cells in the spleens of infected mice. Two-tailed Student’s *t* test was performed for statistical analysis in **B** and **C**. One-way ANOVA, followed by multiple Tukey tests, was performed for statistical analysis for **D**–**G**. Data are representative of 2 independent experiments (*n* = 5 mice/group). *P < 0.05, **P < 0.005, ***P < 0.0005.

Above results clearly demonstrate that PTL coimmunization enhanced protective immune responses following BCG vaccination. To further reveal the antigen specificity and protective function of T cell responses generated by BCG-PTL coimmunization, we carried out the adoptive transfer of CD4^+^ and CD8^+^ T cells isolated from the lungs of BCG, PTL, and BCG-PTL–coimmunized mice to check for their antigen-specific protective response in naive mice. CD4^+^ (1 × 10^6^) and CD8^+^ T cells (1 × 10^6^) were transferred into γ-irradiated Thy1.1 mice followed by a low-dose aerosol challenge of *M.tb* H37Rv. Twenty-five days after infection, mice were sacrificed for CFU enumeration and immune profiling (Figure 8A). We found significant decrease of bacterial load in the mice that received T cells from BCG-PTL–coimmunized animals as compared with the BCG-vaccinated group (Figure 8, B and C). Furthermore, the immune profiling revealed a significant increase in the percentage of INF-γ–producing CD4^+^ and CD8^+^ T cells, with no difference in IL-17–producing T cells in the spleens of mice that received T cells from BCG-PTL–coimmunized animals (Figure 8, D–G). Therefore, T lymphocytes (CD4^+^ and CD8^+^ T cells) isolated from coimmunized mice successfully transferred the protective immunity against TB in naive animals.

### BCG-PTL coimmunization protects antibiotic-treated animals against disease recurrence.

**Figure 9:**
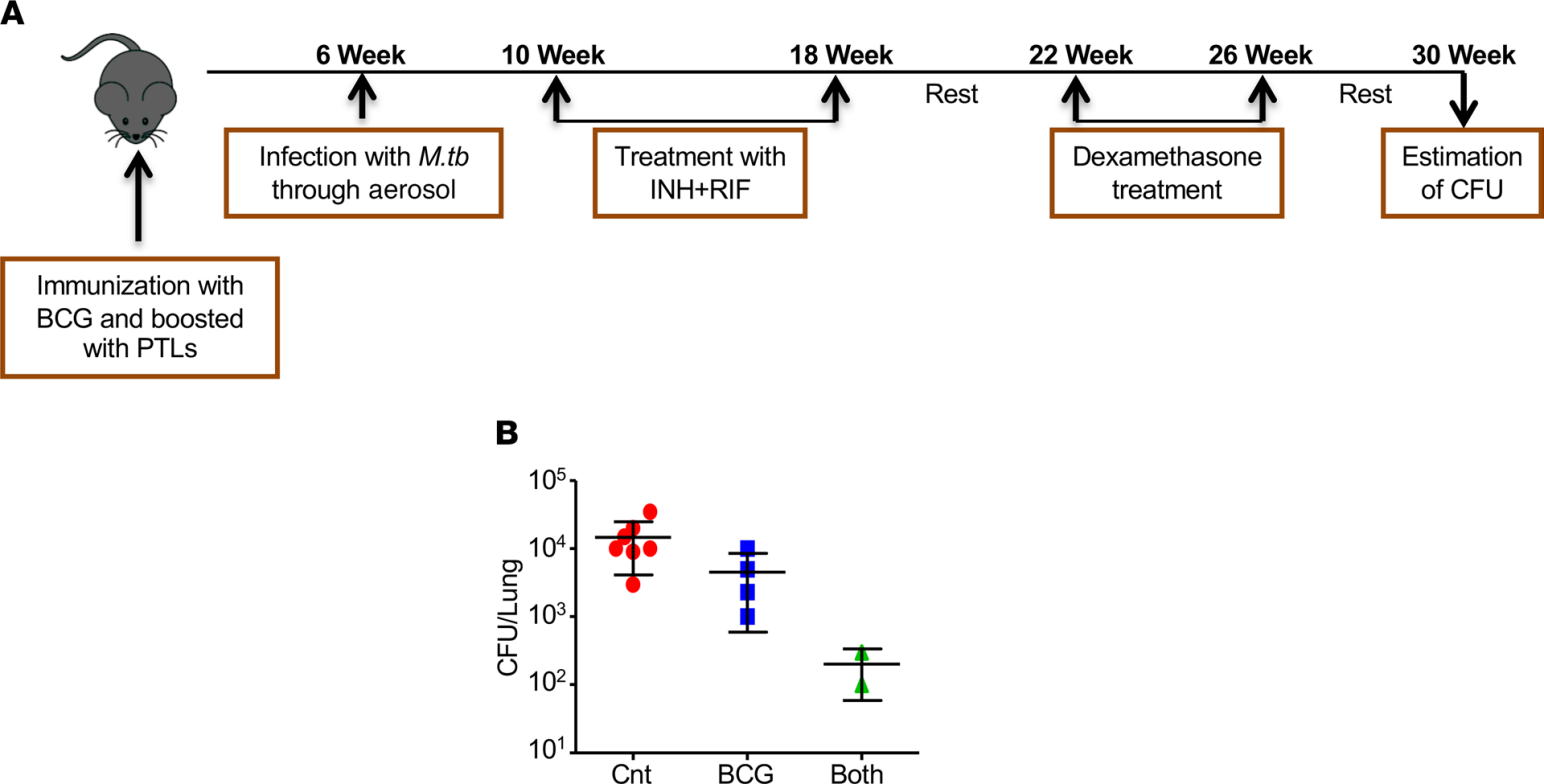
PTL immunization reduces the recurrence of DOTS-associated disease relapse. (**A**) Mice coimmunized with PTL and BCG were infected with H37Rv *M.tb*, followed by treatment with isoniazid and rifampicin for 16 weeks. After 30 days of rest, these mice were treated with dexamethasone for 30 days, followed by 1 more period of rest for 30 days. Mice were then sacrificed for CFU estimation to determine the rate of relapse after treatment. (**B**) CFU from the lung homogenates of the mice. The reactivation experiment was done once with 10 mice in each group.

From the above experiments, it is clear that PTL coimmunization enhances the BCG-induced host-protective immunity, and selectively induces central memory T cell responses, which generally results in long-term protection against TB. To further determine the extent of long-term protection induced by PTL coimmunization, we performed reactivation experiments in the mouse model of TB (Figure 9A). Reactivation rate was calculated as the number of mice reactivated out of the total number of mice in that group. The reactivation results showed that nonvaccinated mice receiving isoniazid (INH) and rifampicin (RIF) treatment exhibited greater disease reactivation (7 of 10 mice, 70%) upon dexamethasone treatment, while mice immunized with BCG showed around 40% (4 of 9 mice) relapse (Table 1). The relapse rate was significantly lower in mice coimmunized with BCG and PTL (2 of 10 mice, 20%) (Table 1). However, there were no differences in terms of bacterial burden in the mice that experienced reactivation (Figure 9B). Since effective memory equates to enhanced protection from primary as well as secondary infections, these observations demonstrate that enhanced proinflammatory responses and Tcm responses induced by BCG-PTL coimmunization might translate into reduced relapse incidents due to reactivation, thus effectively promoting sterile immunity.

## Discussion

The ability of host immune response to mount an activated antigenic T cell response in the case of pathogenic insult is a must to decrease the pathology associated with the infection. While the knowledge about the exact kind of immune responses mounted by the host in the case of TB is still expanding, the role of IFN-γ–producing Th1 has been well documented (29–32). Recent work by several labs, including ours, has shown a synergistic role of Th1 and Th17 cells in mounting potent protective responses against TB (8, 33, 34). The mycobacterial secretory proteins play a crucial role in the induction of protective immune responses during TB infections. Mycobacterial proteins like MPT70, Ag85B, and ESAT6 have already been used as candidates for antigenic vaccines against TB (14, 35, 36). Their success in T cell proliferation, along with IFN-γ assays, have made them promising candidates to be considered in the race for novel subunit vaccines against TB, owing to their promiscuous nature (14, 35, 36). We screened several *M.tb* antigenic peptides for their ability to induce IFN-γ and IL-17 and narrowed it down to 7 overlapping peptides from Ag85B and ESAT6 (Figure 1). The evidence gathered over a period of time indicates the inability of the BCG vaccine in inducing an optimal T cell response against several T cell epitopes harboring immunogenic antigens (37). Also, the BCG vaccine is rendered relatively ineffective in cases of adult pulmonary TB due to its inability to evoke an optimum protective T cell response in the lungs (the primary site of infection) since peripheral T cells have limited influence in the lungs (37). Thus, our rationale was to improve the vaccine’s immunogenic repertoire by including relevant fragments of *M.tb* antigens that might generate an improved response vaccine against TB.

Conventionally *M.tb* produces an array of pathogen-associated molecular patterns (PAMPs), including lipoarabinomanan, phenolic glycolipids, phosphatidylinositol mannosidase, and other lipoproteins. These molecular patterns are recognized by TLRs. TLRs are innate cytosolic surveillance sensors found on professional antigen-presenting cells (APCs) such as macrophages and DCs (18, 38). Interestingly, ligation of these PAMPs is known to trigger both protective as well as pathogenic immune responses (18, 38). Moreover, mice lacking MyD88, a major adapter molecule required for downstream signaling events by the majority of TLR/IL-1R family members, demonstrate enhanced susceptibility to aerosol infection with *M.tb* (39). Several groups have provided convincing reports that TLR2 and TLR9 both are indispensable in protection against TB (17, 40–42). Engagement of these TLRs leads to the activation of a spectrum of transcription factors that induce several proinflammatory cytokines, including IFN-γ, which confers protective immunity against TB. Therefore, we included PamCysSK-4 as the TLR2 agonist and CpG ODN as the TLR9 agonist in our vaccine design. These TLR agonists, along with the pool of 7 overlapping *M.tb* peptides, were packaged in the liposomes for intranasal delivery into the lungs (Figure 4A). Impressively, coimmunization of BCG and PTL not only reduced the bacterial burden (Figure 4) but also led to the increase in the percentage of CD4^+^ and CD8^+^ T cells (Figure 5) that actively participate in providing protective immunity against TB. We also observed enhanced activation of these T cell subsets. In this study, we have analyzed the expression of CD69 and CD25 as early and late T cell activation markers. However, CD25^+^ T cells expressing Foxp3 represent Tregs that have an inhibitory role in the T cell activation. This warrants the analysis of other T cell activation markers. In our study, we also observed a significant increase in the percentage of polyfunctional CD4^+^ and CD8^+^ T cells producing more than 2 cytokines in the mice coimmunized with BCG and PTL (Figure 6). Moreover, our study also provides strong evidence in favor of the antigen-specific nature of these responses from adoptive transfer experiments, where T cells from coimmunized mice were able to impart protective immunity to congenic TB-unexposed naive mice upon *M.tb* infection (Figure 8).

Tcm cells, the perpetual source of Tem cells, dictate the recall responses and are considered indispensable toward potent vaccine response providing long-lasting protective immunity (22, 43). Recently, superior host protection by ΔureC::hly BCG strains has been attributed to its induction of an enhanced Tcm response (11). Interestingly, Tcm were found to be elevated during coimmunization of PTL, along with BCG (Figure 7). FOXO1 plays an important role in establishing long-lived T cell memory responses. BCG-PTL coimmunization reduced the phosphorylation of FOXO1 in the splenocytes of infected animals, which leads to its increased localization in the nucleus, thereby enriching the protective T cell memory responses responsible for enhanced efficacy of vaccine (27, 28). Furthermore, the increase in NF-κB activation in the coimmunized group corroborated with studies crediting NF-κB activation in inducing proinflammatory cytokine production during TB (Supplemental Figure 3) (44–46).

Transcriptome studies on Pep-DCs and stimulated T cells revealed that peptide pool induces differential expression of genes belonging majorly to the immune-relevant signaling pathways, such as JAK/STAT, TNF, TLR, NF-κB, MAPK, and TGF-β. The JAK/STAT pathway is well known to regulate T cell polarization, and deregulation of the JAK/STAT pathway leads to increased susceptibility during TB (47, 48). Similarly, loss of TNF signaling causes increased mortality due to increased bacterial burden and necrotic death of overladen macrophages and granuloma breakdown (49, 50). Moreover, patients receiving TNF-neutralizing therapy have an increased rate of reactivation of latent TB (51). NF-κB has been shown to be critical for the expression of many proinflammatory cytokines required for the protection against TB (45, 52), since NF-κB–KO mice succumb to *M.tb* infection (45, 53). Our data also indicate that peptide pool induces MAPK signaling pathways that have a phenomenal role during TB (54). Dephosphorylation of MAPK, ERK, and P38 leads to increased susceptibility during TB (55). There are many reports suggesting that the MAPK pathway is not only involved in many aspects of immune responses, from initiation of innate immunity to adaptive immunity, but also in its termination through apoptosis and maintenance of T cell homeostasis (56–58). Moreover, MAPKs phosphorylate and activate downstream molecules, resulting in T cell activation, proliferation, and differentiation into T helper phenotypes. *M.tb*-induced production of proinflammatory cytokines also depends on MAPK activation (57, 58).

In spite of the limited ability of the BCG vaccine to provide protective immunity against adult pulmonary TB, it is quite effective in mounting a strong protective response in young children against meningeal and other disseminated TB. Considering that a large percentage of the population in countries with high TB burden are BCG vaccinated at birth, an improvised strategy like ours that accentuates BCG efficacy by selectively increasing Tcm cell pools and polyfunctional T cells, which in turn provide long-lasting immune response against TB, is long desired. The study warrants further validation in TB models more similar to humans, such as nonhuman primates.

## Methods

### Mice.

All C57BL/6 mice (6–8 weeks of age) were maintained in the animal facility of the ICGEB and provided for experiments as and when required.

### Generation of DCs.

C57BL/6 mice were euthanized, and the femurs were isolated. BM was flushed out with RPMI 1640 medium using a 2.0 mL syringe (26.5 gauge). The cells were washed twice with PBS and then cultured in complete RPMI 1640 medium (Invitrogen) supplemented with GM-CSF (40 ng/mL) and IL-4 (10 ng/mL) on 12-well plates (1 million cells/mL). On the third day, 75% of the medium was replaced with fresh DC culture medium. On day fifth day, the suspended cells were removed, and the loosely adherent cells were collected as immature DCs (CD11c^+^ cells were > 90%). For mature DCs, immature DCs were stimulated with LPS (1 μg/mL) for 24 hours. FACS analysis using anti-CD11c, -CD80, -CD86, and –MHC class II antibodies suggested that > 90% of the cells were conventional DCs. DCs were either left untreated or treated overnight with 20 μg/mL of CSA or 0.2 μg/mL of each peptide, followed by coculture with CD3^+^ T cells isolated from *M.tb*-infected and DOTS-treated animals for 48 hours.

### Human PBMC isolation.

Blood samples collected from PPD^–^ and PPD^+^ BCG–vaccinated healthy individuals were diluted in DPBS (Gibco, 14190250) at a ratio of 1:2 and layered onto Ficoll-Paque Plus (catalog GE17-1440-02) followed by centrifugation at 500*g* for 35 minutes at 25°C. Out of the 4 layers, the uppermost plasma was removed by pipette, and the second layer of the cells containing PBMCs was gently removed and suspended in complete DMEM (Invitrogen). These cells were then pelleted, counted, and seeded in the 12-well plates for further experiments.

### M.tb infection of mice and estimation of CFU.

*M.tb* H37Rv and BCG cultures were grown in 7H9 (Middlebrooks, Difco) medium supplemented with 10% OADC (oleic acid, albumin, dextrose, and catalase; Difco) and with 0.05% Tween 80 and 0.5% glycerol, and cultures were grown to mid-log phase. Aliquots of the cultures in 20% glycerol were preserved at –80°C, and these cryopreserved stocks were used for infections.

Mice were infected with H37Rv via the aerosol route using a Madison aerosol chamber (University of Wisconsin, Madison, Wisconsin, USA) with its nebulizer precalibrated to deposit around of 150 bacilli to the lungs of each mouse as previously described (8). Briefly, bacterial stocks were recovered from the freezer and quickly thawed and subjected to light ultrasonication to obtain a single cell suspension. A total of 15 mL of the bacterial cell suspension (10 × 10^6^ cells per mL) was placed in the nebulizer of the Madison aerosol chamber precalibrated to deliver the desired number of CFUs to the lungs of animals placed inside the chamber, via the aerosol route. At day 1 after infection, 3 randomly selected mice were sacrificed, lungs were harvested and homogenized in 0.2 μm filtered PBS, and neat samples (without any dilution) were plated onto 7H11 Middlebrooks (Difco) plates containing 10% OADC (Difco). Neat, 10-fold diluted and 100-fold diluted lung, liver, and spleen cell homogenates were plated in triplicate on the 7H11 plates and incubated at 37°C for 21 to 28 days for the organs harvested at different time points. Colonies were counted, and CFU was calculated accordingly. Mice from various groups were euthanized at the indicated time points in various experiments; their organs were harvested for obtaining CFU counts and/or immune cell subpopulations for immunological studies as described under other subsections.

### Antibiotic treatment.

Thirty days after infection, groups of mice were treated with 10 mg/kg of RIF and 10 mg/kg of INH (MilliporeSigma) administered in the drinking water (changed daily) for 12 weeks. *M.tb*-infected control mice received plain drinking water.

### Isolation of T cell lymphocytes from M.tb-infected animals.

Lungs and spleens from infected animals were harvested and washed by swirling in PBS. They were opened up by cutting longitudinally and then cut into 0.5 cm pieces. These lung pieces were agitated in 25 mL of extraction buffer (PBS, 3% FCS, 1 mM dithiothreitol, 1 mM EDTA) for 30 minutes at 37°C. This slurry was passed through a loosely packed nylon wool column to remove the aggregates. The filtrate was layered on a discontinuous Percoll gradient (Amersham Pharmacia Biotech). This gradient was then centrifuged at 252*g* for 20 minutes at 25°C. Cells at the interface were collected and washed in staining buffer (PBS, 3% FCS). Spleen cells were homogenized and washed with RBC lysis buffer to remove RBCs. Cells from the lungs and spleens were cultured for surface and intracellular staining as described in the subsection.

### Preparation of PTL.

Lipid mixtures received from MilliporeSigma (catalog L4395) were used for the preparation of liposomes, which can encapsulate a broad spectrum of hydrophilic and amphipathic molecules of the low, medium, and high molecular weight (including peptides, proteins, and oligo- and polynucleotides). We mixed the *M.tb* peptides with lipid mixtures, along with TLR2 and TLR9 ligands Pam3Cys-SK-4 (catalog ALX-165-066-M002, Enzo Life Sciences) and CpG ODN (catalog ALX-746-003-C100, Enzo Life Sciences), respectively, as per manufacturer’s protocol to get the homogeneous mixture of peptides and TLR ligands with liposomes (1 mL of PTL mixture contains 10 μg of each peptide, 100 μg of Pam3Cys-SK-4, and 10 μg of CpG ODN). Then, these mixtures were injected intranasally into the mice (50 μL per mice per dose). To confirm the successful delivery of the liposomes into the lungs, the liposomes were stained with PKH67 dye (MilliporeSigma) as per manufacturer’s protocol. Sections of the lungs were seen under microscope for the fluorescence of the dye.

### Immunization.

Mice were immunized with (a) BCG (s.c.) (1 × 10^6^ bacteria), (b) PTL (intranasal), (c) BCG (s.c.) + PTL (Intranasal), and (d) vector only. Mice were subsequently rested for 21 days and then challenged with *M.tb* strain H37Rv by the aerosol route. Organs like lungs, livers, and spleens were harvested for determination of bacterial burden and profiling of immune memory cell responses at different days after infection.

### Histology.

Lung tissues were fixed in formalin solution and coated with wax for sectioning. Sections were stained with H&E dyes, and slides were placed under a microscope. Granulomas were analyzed to obtain the granuloma score.

### Flow cytometry.

Spleens and lungs were isolated from respective mice and macerated by frosted slides in ice-cold RPMI 1640 (Invitrogen) containing 10% FBS to prepare a single cell suspension. RBCs were lysed with RBC cell lysis buffer, incubated at room temperature for 2–3 minutes, and washed with RPMI 1640 containing 10% FBS. The cells were counted, and 1 × 10^6^ cells were used for surface staining. For intracellular staining 1 × 10^6^, cells were cultured per well in 12-well plates (Tarsons) in the presence of H37Rv CSA overnight. Subsequently, 0.5 μg/mL Brefeldin A and 0.5 μg/mL of Monensin solution (BioLegend) were added during the last 4 hours of culture. Cells were then washed twice with FACS buffer (PBS + 3% FCS) and stained with antibodies directed against surface markers. After staining, cells were washed again with FACS buffer and fixed with 100 μL fixation buffer (BioLegend) for 30 minutes, washed, and resuspended in 200 μL permeabilization buffer (BioLegend) before being stained with fluorescently labeled anti-cytokine antibodies. FACS Verse BD was used for acquiring the cell population, and data analysis was done using Flow Jo (Tree Star Inc.).

### Antibodies and reagents.

We used the following BioLegend antibodies: anti-CD3 (clones 17A2 and HIT3a) Pacific-blue, FITC, PE, or APC; anti-CD4 (clones GK1.5 and A161A1) FITC, PE, PerCP-Cy5, PE/Cy7, or APC; anti-CD8 (clone 53-6.7) FITC, APC/Cy7, or APC; anti-CD44 (clone IM7) FITC; anti-CD62L (clone MEL-14) APC; anti-CD25 (clone 3C7) APC; anti-CD69 (clones H1.2F3 and FN50) FITC or PE; anti-CD197 or -CCR7 (clone 4B12) PE/Cy7; anti–IFN-γ (clones XMG1.2 and 4S.B3) APC or PE; anti–IL-12 (clone C15.6) PerCP/Cy5.5; anti–IL-17 (clone TC11-18H10.1) PE-Cy-7; anti–IL-2 (clone JES6-5H4) FITC; anti–TNF-α (clone MP6-XT22) PE or PerCP/Cy5.5; and anti–TGF-β (clone TW7-16B4) APC. Brefeldin A Solution (1000×), Monensin Solution (1000×) (catalog 420701), and Intracellular Staining Permeabilization Wash Buffer (10×) were purchased from BioLegend.

### T cell adoptive transfer.

For adoptive transfer experiments, T cells from lungs of the infected mice that belonged to 3 groups — (a) BCG immunized, (b) PTL immunized, and (c) BCG-PTL–coimmunized — were isolated and sorted into CD4^+^ and CD8^+^ T cells. CD4^+^ (1.0 × 10^6^ cells/mouse) and CD8^+^ T cells (1.0 × 10^6^ cells/mouse) were adoptively transferred into γ-irradiated Thy1.1 mice. Recipient mice were challenged with H37Rv through the aerosol route for the determination of bacterial burden and immune responses in the lungs of respective mice.

### Mouse model of TB reactivation.

Mice infected with *M.tb,* with low-dose aerosol infection model, were treated with 10 mg/kg of INH and RIF administered ad libitum (in the drinking water) or treated to mice coimmunized with BCG and PTL or immunized with BCG alone for 12 weeks starting at the fourth week after infection. These mice were then rested for 30 days, followed by treatment with dexamethasone (5 mg/kg, i.p.) 3 times per week for 30 days. These mice were again rested for next 30 days. Ten mice from each group were then sacrificed, and CFUs were estimated from lung homogenates to determine the reactivation rate of *M.tb*.

### RNA-Seq.

Total RNA was isolated from Un-DCs and DCs pulsed with peptide pool (Pep-DC), as well as from unstimulated cocultured T cells (Un-TCs) and cocultured T cells (Pep-TCs), along with DCs pulsed with peptide pool using an RNeasy RNA isolation kit (Qiagen). The raw reads generated for Un-DCs and Pep-DCs and unstimulated cocultured T cells and Pep-TCs were subjected to quality check using FastQC (version 0.11.5, http://www.bioinformatics.babraham.ac.uk/projects/fastqc/), using parameters like base quality score distribution, sequence quality score distribution, average base content per read, and GC distribution in the reads. Illumina Adapter (AGATCGGAAGAGC) was removed using Trim Galore (version 0.4.1), a wrapper script to automate quality and adapter trimming, as well as quality control. Clean reads were mapped on reference Mus-musculus_GRCm38.p6 using TopHat v2.1.0. Differential analysis was performed on counted mapped reads in a range of positions on a chromosome for Un-DC versus Pep-DC and for Un-TC versus Pep-TC combinations, predicted using htseq-count software; then, differential gene expression was found using DeSeq, a Bioconductor R package that estimates variance-mean dependence in count data and implements a range of statistical methodology based on the negative binomial distributions. Functional annotation for combination was performed using UniProt database and DAVID. The transcriptome data are available on Gene Expression Omnibus (https://www.ncbi.nlm.nih.gov/geo/query/acc.cgi?acc=GSE164258; accession no. GSE164258).

### Western blot.

Spleens were harvested from all experimental groups, homogenized, and made up the cell lysate. Whole cell lysate was prepared by using lysis buffer (50 mM Tris-HCl [pH 7.4], 5 mM EDTA, 120 mM NaCl, 0.5% Nonidet P-40, 0.5 mM NaF, 1 mM dithiothreitol, 0.5 mM phenylmethylsulfonyl fluoride) along with HALT phosphatase inhibitor mixture (78420, Thermo Fisher Scientific) and protease inhibitor mixture (78410, Thermo Fisher Scientific) for 1 hour. Samples were electrophoresed on a 10% SDS-polyacrylamide gel and electroblotted onto polyvinylidene difluoride membranes. Blots were blocked for 1 hour in 5% BSA in PBS with 0.1% Tween 20. NF-κB, pNF-κB, FOXO1, and pFOXO1 proteins were detected with NF-κB (8242S), pNF-κB (3033S), FOXO1 (2880S), and pFOXO1 (9461S) monoclonal antibodies, respectively, at a dilution of 1:250 and as recommended by the manufacturer (Cell Signaling Technology). Goat anti–rabbit IgG G-conjugated horseradish peroxidase (sc-2004) (diluted 1:5000) was used as a secondary antibody (Santa Cruz Biotechnology Inc.). Immunoblotting for β-actin was carried out to confirm equal loading.

### Statistics.

Statistical analyses were conducted using GraphPad Prism software by performing 2-tailed Student’s *t* test or 1-way ANOVA, followed by multiple Tukey tests. *P* < 0.05 was accepted as an indication of statistical significance. *P < 0.05, **P < 0.005, ***P < 0.0005.

### Study approval.

Animal experiments were performed as per ethical guidelines approved by the Institutional Animal Ethics Committee held in July 2015 at the ICGEB (New Delhi, India) and the Department of Biotechnology guidelines (Government of India) (approval ID, ICGEB/AH/2015/01/IMM-45). All mice used for experiments were ethically sacrificed by asphyxiation in carbon dioxide according to the institutional and Department of Biotechnology (DBT), Government of India, regulations. The human studies were ethically approved (approval ID, 359/SHRMU/19/2010/07) by the Institutional Human Ethics Committee, Jawaharlal Nehru University, New Delhi, India, and Regional Medical Research Centre (RMRC), Odisha, India.

## Author contributions

SK and VPD initially started the study. SK, AB, CS, SRK, DKS, SC, and VPD performed in vitro experiments and analyzed data. SK, AB, and VPD performed all animal experiments. SRK also assisted in animal experiments. GP performed experiments with human PBMCs. VPD conceived the hypothesis and supervised the experiments. AB and VPD designed experiments and analyzed the data. GD provided the resources and edited the manuscript. AB and VPD wrote the manuscript.

## Supplementary Material

Supplemental data

## Figures and Tables

**Table 1:**
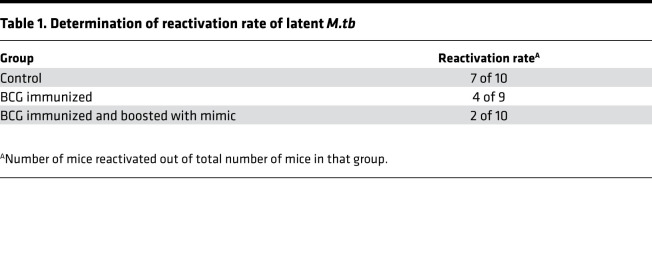
Determination of reactivation rate of latent *M.tb*

## Version 1 01/14/2021

In-Press Preview

## Version 2 02/22/2021

Electronic publication
