# Supplementary material for: Intranasal immunization with peptide-based immunogenic complex enhances BCG vaccine efficacy in a murine model of tuberculosis
Source: JCI Insight. 2021 Feb 22;6(4):e145228. doi: 10.1172/jci.insight.145228 (PMC7934935; doi:10.1172/jci.insight.145228)
Supplement: Supplemental data [file jciinsight-6-145228-s157.pdf]

Supplementary Materials:

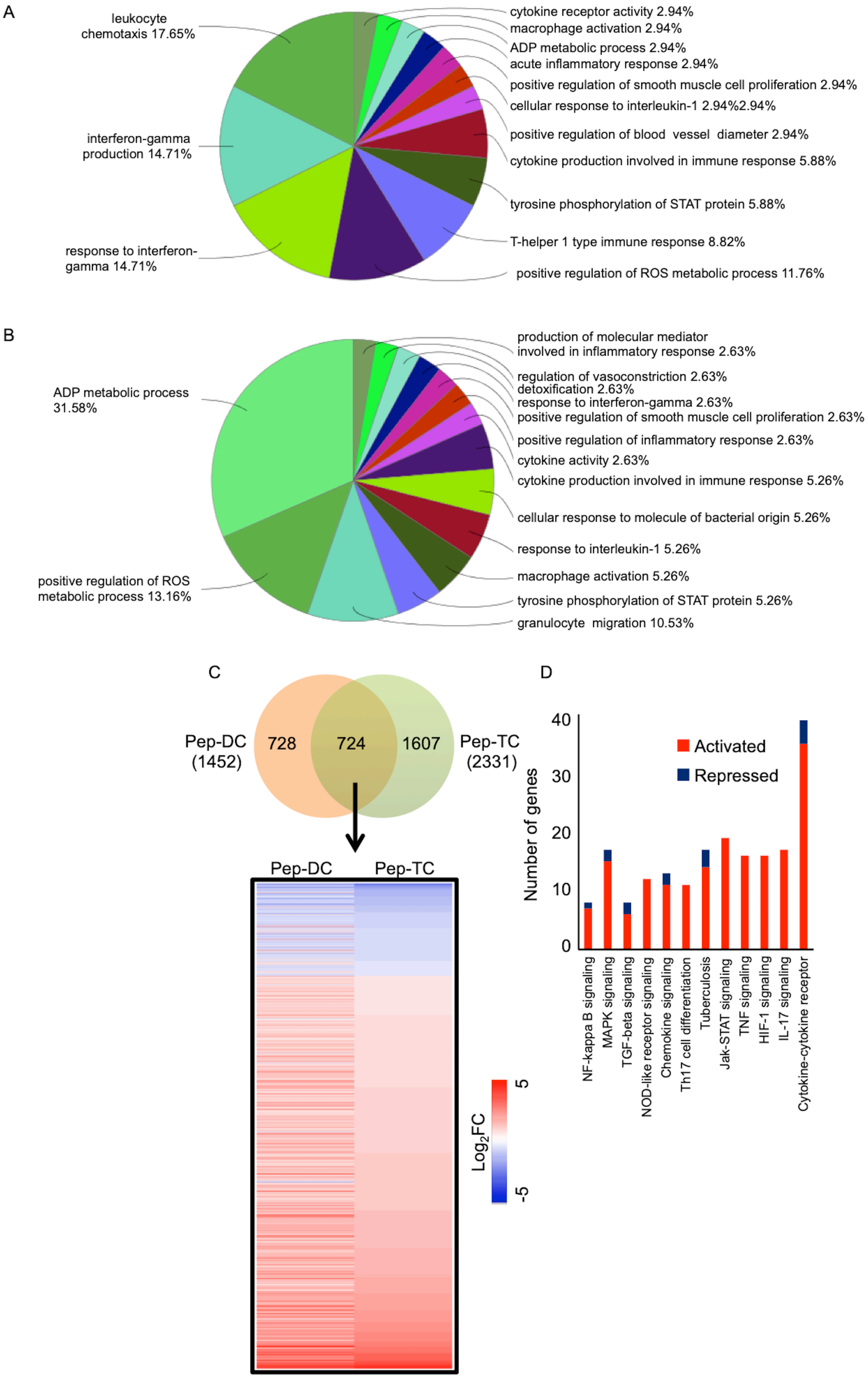

Supplementary Figure 1: *M.tb* peptides induce upregulation of genes that confer protection against TB in both DCs and T cells. (A) Pie diagram indicating the GO biological processes upregulated in DCs pulsed with peptide pool as compared to unstimulated DCs. **(B)** Pie chart indicating the percentage of genes belonging to different GO biological processes in stimulated T cells as compared to unstimulated T cells. **(C)** Heatmap depicting the expression profile of the genes common between the DCs and T cells. **(D)** KEGG pathway analysis of the shared genes representing the significantly affected pathways. **Un-DC**: unstimulated DCs, **Pep-DC**: DCs pulsed with peptide pool, **Un-TC**: T cells co-

cultured with unstimulated DCs, **Pep-TC**: T cells co-cultured with DCs pulsed with peptide pool. RNAseq was performed once in triplicates (n=3).

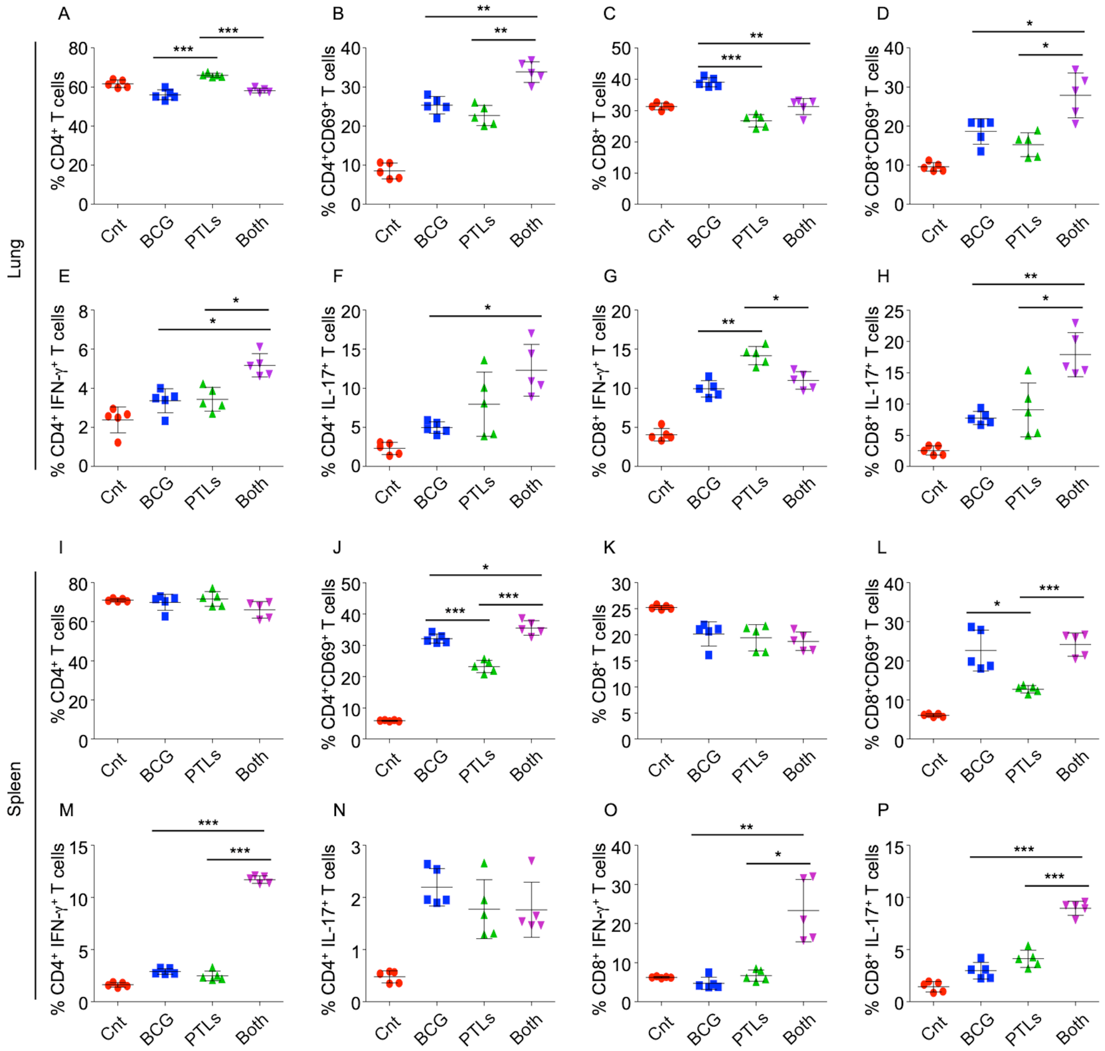

Supplementary Figure 2: Pre-challenge immune responses in the lungs and the spleen of vaccinated mice. (A-H) T lymphocytes were isolated from the lungs of all experimental groups and stained with 7AAD, anti-CD3, anti-CD4, anti-CD8, anti-CD69, anti-IFN-γ and anti-IL-17 antibodies. Percentage of CD4$^+$ T cells **(A),** CD4$^+$CD69$^+$ T cells **(B)**, CD8$^+$ T cells **(C)** and CD8$^+$CD69$^+$ T cells **(D)** in the lungs of unvaccinated (Cnt) and vaccinated animals. **(E-H)** Expression of IFN-γ and IL-17 on CD4$^+$ and CD8$^+$ T cells in the lungs. **(I-P)** T lymphocytes isolated from the spleen of all experimental groups were stained with 7AAD, anti-CD3, anti-CD4, anti-CD8, anti-CD69, anti-IFN-γ and anti-IL-17 antibodies. Percentage of CD4$^+$ T cells **(I),** CD4$^+$CD69$^+$ T cells **(J)**, CD8$^+$ T cells **(K)** and CD8$^+$CD69$^+$ T cells **(L)** in the spleen of different groups. **(M-P)** Expression of IFN-γ and IL-17 on CD4$^+$ and CD8$^+$ T cells in the spleen. One-way ANOVA followed by multiple tukey tests was performed for statistical analysis. Data is representative of two independent experiments (n=5 mice/group). *p<0.05, **p<0.005, ***p<0.0005.

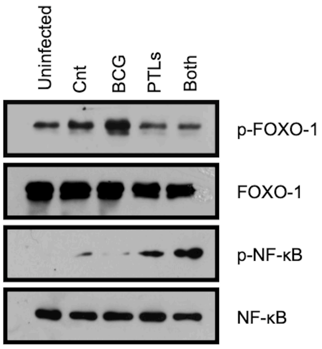

Supplementary Figure 3: Enhanced NF-kB and FOXO-1 activation in the spleen of infected mice co-immunized with BCG-PTLs. Cell lysates prepared from the splenocytes of infected mice from all the experimental groups were used to analyze the phosphorylation status of FOXO-1 and NF-kB by immunoblotting. Data is representative of two independent experiments.

Supplementary Table 1: List of Peptides used in the study

S. No.	Peptide Sequence	Position	Protein name	Rv number
P1	AWGRRLMIGTAAAVVLPG	10aa - 27aa	Ag85B	Rv1886c
P2	TAAAVVLPGLVGLAGGAA	19aa - 36 aa	Ag85B	Rv1886c
P3	WDINTPAFEWYYQSGLSI	91aa - 108aa	Ag85B	Rv1886c
P4	LDEGKQSLTKLAAAW	29aa - 43aa	ESAT6	Rv3875
P5	KQSLTKLAAAWGGSG	33aa - 47aa	ESAT6	Rv3875
P6	TKLAAAWGGSGSEAY	37aa - 51aa	ESAT6	Rv3875
P7	LARTISEAGQAMASTEGNVTGMFA	72aa - 95aa	ESAT6	Rv3875
P8	AVAASNNPELTTLTAALSGQLNPQV	61aa-85aa	Mpt70	Rv2875
P9	ALSGQLNPQVNLVDTLNSGQYTVFA	76aa-100aa	Mpt70	Rv2875
P10	FSKLPASTIDELKTNSSLLTSILTY	106aa-130aa	Mpt70	Rv2875
P11	GNADVVCGGVSTANATVYMIDSVLM	166aa-190aa	Mpt70	Rv2875
P12	TVYMIDSVLMPPA	181aa-193aa	Mpt70	Rv2875
P13	AVDAADKVLGYRNWL	278-292aa	GlcB	Rv1837
P14	KVLGYRNWLGLNKGD	284-298aa	GlcB	Rv1837

Supplementary Table 2: List of seven antigenic peptides used for further studies. Experiments from A-C were repeated with the pool of seven peptides.

Peptides	Sequences
P-1	AWGRRLMIGTAAAVVLPG
P-2	TAAVVLPGLVGLAGGAA
P-3	WDINTPAFEWYYQSGLSI
P-4	KQSLTKLAAAWGGSG
P-5	TKLAAAWGGSGSEAY
P-6	LDEGKQSLTKLAAAW
P-7	LARTISEAGQAMASTEGNVTGMEA
